# Ultra-low cost eyetracking as an high-information throughput alternative to BMIs

**DOI:** 10.1186/1471-2202-12-S1-P103

**Published:** 2011-07-18

**Authors:** William W Abbott, A Aldo Faisal

**Affiliations:** 1 Department of Bioengineering, Imperial College London, London, SW7 2AZ, UK; 2 Department of Computing, Imperial College London, London, SW7 2AZ, UK

## 

Advancement in Brain machine interfaces (BMIs) holds the hope to restore vital degrees of independence for patients with high-level neurological disorders, improving their quality of life while reducing their dependency on others [1]. Unfortunately these emerging rehabilitative methods come at considerable clinical and post-clinical operational costs, beyond the means of the majority of patients [1]. Here we consider an alternative: eye movements. Eye movements provide a feasible alternative BMI basis as they tend to be spared degradation by neurological disorders such as Muscular dystrophy, high-level spinal injuries and multiple sclerosis because the Occululomotor system is innervated from the midbrain rather than the spinal column. Eye tracking and gaze based interaction is a long established field, however cost, accuracy and effective system integration have meant these systems are not widely used. We have developed an ultra-low cost 3D gaze-tracker based on mass market video game equipment that matches the performance of commercial systems 500 times as expensive.

We developed a calibration method for 3D eye gaze, that requires only a standard computer monitor as opposed to 3D equipment, no information on eye geometry, and allows free head movement following calibration. Our method enables us to track eye movements off the computer screen, e.g. to drive a wheel chair or the end-point of a prosthetic arm. Unlike other BMIs, training is virtually unnecessary as the control intention can be taken from natural eye movements. By tracking both eyes, a significantly higher information rate can be obtained both by making 2D gaze estimates more accurate and by adding another spatial dimension in which to make commands. Our ultra-low cost 3D eye tracking system operates at below 50 USD material cost with 0.5-1 degree resolution at 120 Hz sample rate, we achieved this by reverse engineering mass-marketed video console components. This high-speed accuracy allows us to drive a mouse cursor at this performance level in real-time at a data rate of over 1100 bits/s, while capturing depth allows us, in theory to drive a continuous control user interface at a multiple of that rate. These information rates are orders of magnitude times higher than other BMI approaches e.g. non-invasive cortical methods (maximum 1.63 bits/sec) such as Electroencephalography (EEG), Magnetoencephalography (MEG), functional Magnetic Resonance imaging (fMRI), and Near Infrared Spectroscopy (NIRS); Cortical Invasive methods (maximum 3.30 bits/sec) such as MEAs and EcOG and non-cortical non-invasive methods (maximum 6.13 bits/sec) such as EMG, Speech Recognition and Mechanical Switches[1,2]. Thus, our delay free, real-time control is suitable to allow paralyzed patients to play live action video games, as we recently demonstrated to the public[3]. Moreover, the novel approach of using 3D point of gaze makes the users interaction with their surroundings more intuitive, without detracting from the eye’s sensory function. A potential example, in a wheelchair context, is that the user simply looks where they would like to drive. With this low cost device and the rich 3D gaze information we provide an extremely intuitive and non-intrusive BMI alternative that can be developed that is economically accessible to patients in the developed and the developing world.
